# Subjective evidence evaluation survey for many-analysts studies

**DOI:** 10.1098/rsos.240125

**Published:** 2024-07-24

**Authors:** Alexandra Sarafoglou, Suzanne Hoogeveen, Don van den Bergh, Balazs Aczel, Casper J. Albers, Tim Althoff, Rotem Botvinik-Nezer, Niko A. Busch, Andrea M. Cataldo, Berna Devezer, Noah N. N. van Dongen, Anna Dreber, Eiko I. Fried, Rink Hoekstra, Sabine Hoffman, Felix Holzmeister, Jürgen Huber, Nick Huntington-Klein, John Ioannidis, Magnus Johannesson, Michael Kirchler, Eric Loken, Jan-Francois Mangin, Dora Matzke, Albert J. Menkveld, Gustav Nilsonne, Don van Ravenzwaaij, Martin Schweinsberg, Hannah Schulz-Kuempel, David R. Shanks, Daniel J. Simons, Barbara A. Spellman, Andrea H. Stoevenbelt, Barnabas Szaszi, Darinka Trübutschek, Francis Tuerlinckx, Eric L. Uhlmann, Wolf Vanpaemel, Jelte Wicherts, Eric-Jan Wagenmakers

**Affiliations:** ^1^ Department of Psychology, University of Amsterdam, Amsterdam, The Netherlands; ^2^ Utrecht University, Utrecht, The Netherlands; ^3^ Institute of Psychology, ELTE Eötvös Lorénd University, Budapest, Hungary; ^4^ Heymans Institute for Psychological Research, University of Groningen, Groningen, The Netherlands; ^5^ Allen School of Computer Science and Engineering, University of Washington, Seattle, WA, USA; ^6^ Hebrew University of Jerusalem, Jerusalem, Israel; ^7^ Institute for Psychology, University of Münster, Münster, Germany; ^8^ Center for Depression, Anxiety and Stress Research, McLean Hospital, Belmont, MA, USA; ^9^ Department of Business, University of Idaho, Moscow, ID, USA; ^10^ Stockholm School of Economics, Stockholm, Sweden; ^11^ Department of Psychology, Leiden University, Leiden, The Netherlands; ^12^ Department of Statistics, Ludwig-Maximilians-Universität München, Munchen, Bayern, Germany; ^13^ University of Innsbruck, Innsbruck, Tirol, Austria; ^14^ Seattle University, Seattle, WA, USA; ^15^ Meta-Research Innovation Center at Stanford (METRICS) and Departments of Medicine, of Epidemiology and of Population Health, of Biomedical Data Science, and of Statistics, Stanford University, Stanford, CA, USA; ^16^ University of Conneticut, Storrs, CT, USA; ^17^ University Paris-Saclay, Gif-sur-Yvette, France; ^18^ Vrije Universiteit Amsterdam, Amsterdam, Noord-Holland, The Netherlands; ^19^ Karolinska Institutet, Solna, Sweden; ^20^ ESMT Berlin, Berlin, Germany; ^21^ Department of Statistics and The Institute for Medical Information Processing, Biometry, and Epidemiology, LMU Munich, Munchen, Bayern, Germany; ^22^ Division of Psychology and Language Sciences, University College London, 26 Bedford Way, London WC1H 0AP, UK; ^23^ University of Illinois—Urbana-Champaign, Urbana, IL, USA; ^24^ School of Law, University of Virginia, 580 Massie Road, Charlottesville, VA, USA; ^25^ Max Planck Institute for Empirical Aesthetics, Frankfurt am Main, Germany; ^26^ University of Leuven, Leuven, Belgium; ^27^ INSEAD, Fontainebleau, Île-de-France, France; ^28^ Department of Methodology and Statistics, Tilburg University, Tilburg, The Netherlands; ^29^ Dartmouth College, Hanover, NH, USA; ^30^ Department of Psychiatry, Harvard Medical School, Boston, MA, USA; ^31^ Neurospin CEA, Gif-sur-Yvette, Île-de-France, France; ^32^ The Institute for Medical Information Processing, Biometry, and Epidemiology, LMU Munich, Munchen, Bayern, Germany; ^33^ Nieuwenhuis Institute for Educational Research, University of Groningen, Groningen, The Netherlands

**Keywords:** open science, team science, scientific transparency, metascience, crowdsourcing analysis

## Abstract

Many-analysts studies explore how well an empirical claim withstands plausible alternative analyses of the same dataset by multiple, independent analysis teams. Conclusions from these studies typically rely on a single outcome metric (e.g. effect size) provided by each analysis team. Although informative about the range of plausible effects in a dataset, a single effect size from each team does not provide a complete, nuanced understanding of how analysis choices are related to the outcome. We used the Delphi consensus technique with input from 37 experts to develop an 18-item subjective evidence evaluation survey (SEES) to evaluate how each analysis team views the methodological appropriateness of the research design and the strength of evidence for the hypothesis. We illustrate the usefulness of the SEES in providing richer evidence assessment with pilot data from a previous many-analysts study.

## 1. Introduction

Researchers adopt a wide range of approaches when analysing data, and their equally justifiable choices about statistical procedures, data processing and the inclusions of covariates can affect the conclusions they draw [1,2]. In fields ranging from epidemiology to psychology to economics, concerns have been raised about the robustness of published evidence since researchers find different answers to the same research question with the same data. This uncertainty in the statistical outcomes is not addressed within standard statistical inference practices and usually remains hidden from view when only a single analysis is presented (e.g. [3]), resulting in overconfidence and model myopia [4–7].

The robustness of an empirical claim on the basis of a single (new, preregistered) dataset can be assessed through multiverse or vibration of effects analysis [8,9] and many-analysts approaches [6] (but see [10] for a critical reflection on robustness analyses). These approaches are designed to reveal the range of justifiable analytic decisions and their consequences for the reported outcome. In a multiverse or vibration of effects analysis, different analytic paths are systematically explored by the same analyst(s) (e.g. [9,11–15]). In a many-analysts project (also referred to as ‘crowdsourced analyses’ [6] or a ‘multi-analyst approach’ [4,7]), different independent analysis teams analyse the same dataset (e.g. [16–23]). In both cases, the end result is an evaluation of the consistency of the observed outcomes across all analyses.

Many-analysts projects appear particularly well suited to mitigate arbitrariness of individual analytic choices, while still allowing for expertise-based analytic decisions concerning data preprocessing, variable exclusion, and model specification. By drawing from a pool of plausible analyses, a many-analysts approach thus enables one to quantify variability across teams based on theory-driven analysis choices and plausible statistical models rather than emphasizing just one analyst’s approach. Specifically, if a range of different experts arrive at the same conclusion, we can be fairly confident that the effect is robust. If they reveal a wide variety of outcomes, we need to evaluate why those choices matter.

Many-analysts projects are a recent innovation, but they have already been adopted in many different fields, including neuroscience [16,17,24–26], economics [27,28], epidemiology [29], ecology [30,31], political science [18] and psychology [19–23,32–35]. Many of these projects concluded that different but justifiable analytic decisions led to diverging outcomes, sometimes with statistically significant effects in opposite directions (e.g. [18,28,34], but see [23]).

### 1.1. Beyond effect sizes: acknowledging insights and concerns of analysis teams

The many-analysts approach can reveal the extent to which the reported outcome varies with different, expert-driven analytical decisions. The approach typically focuses exclusively on a single outcome of interest from each team (such as an odds ratio (e.g. [21]) or a standardized beta coefficient; e.g. [23], but see [17]). These effect size estimates are (visually) summarized to provide an overall impression of the results (but see [36,37] for recently proposed alternative statistical approaches).

This exclusive focus on effect size estimates from each team carries several implicit assumptions: (a) the statistical analyses of each team are sufficiently similar so that they can be summarized using a common effect size metric, (b) further insights from the analysis teams are not relevant when measuring the consistency of the reported results, and (c) analysis teams, by participating in the project, fully endorse the quality of the data they are given and the appropriateness of the research design (cf. 10]).

Commentaries on the recently published Many-Analysts Religion Project [23], studying the relationship between self-reported well-being and religiosity, challenge all three assumptions (see also [18,20,38–51]). First some analysis teams applied more complex approaches that did not naturally yield the specified outcome measure (i.e. standardized regression coefficients). These analyses included structural equation modelling machine learning and even multiverse analyses.^1^ Second many teams presented more nuanced interpretations of the primary effect based on sub-group analyses or multivariate approaches which helped determine the conditions under which the hypothesized relation occurred. Third some teams raised concerns about measurement invariance in the data themselves. Others criticized the formulation of the research question an issue that surfaced in the previous many-analysts projects. In sum relying on a single reported effect from each team leaves no room for a more nuanced and detailed interpretation of the results and the underlying data.

### 1.2. Assessment of subjective evidence

Although measuring the distribution of plausible effect sizes can provide important insights about the robustness of an empirical result [36,37], we argue that it is incomplete (see also [51,52]). To reap the full benefits of involving multiple analysts, we should also examine the broader context in which analysts made their choices: their prior beliefs about the effect, their assessment of the adequacy of the design, or the stability of the effect; thus a subjective measure of evidence. Here, we define subjective evidence as the extent to which one believes in the presence of the effect or relationship given the data and study design.

The idea of collecting a subjective assessment of research evidence in a systematic, reflective and standardized manner is uncommon in the quantitative social and behavioural sciences. Perhaps one could view the discussion section of an empirical article as a narrative subjective evaluation of the obtained evidence, as this is typically where authors discuss the limitations and implications of the quantitative results. It would be challenging, however, to include a narrative summary for every team in a many-analysts project. By contrast, the measurement of subjective evidence has been included in previous many-analysts projects, although typically only as single items and not with the intention of capturing different aspects of scientific evidence. For instance, analysts may be asked about the plausibility of the research claim (e.g. [21]) or whether the assumed effect or relation was confirmed by the data (e.g. [23]). Other aspects, such as the stability of the effect or the pertinence of the effect size, remain unexplored. Moreover, while some previous studies included measures regarding the appropriateness of analytic approaches, this was not conducted in a self-reflective manner; typically, the analytic teams assessed each other’s analytic strategy. Finally, analysis teams’ concerns about the research design and data have been raised through personal correspondence [21] and/or commentaries [18,23], but are not systematically addressed in the manuscript itself. Here, we propose a short, simple and systematic assessment of each team’s subjective evaluation of the evidence, design and data.

Other fields commonly use the subjective evaluation of the evidence as a scientific assessment, often to systematically integrate evidence from different studies and sources. In the evaluation of randomized controlled trials and systematic literature reviews, for instance, subjective assessment of evidence is particularly relevant, as objective quantification is difficult. For such reviews, existing guidelines help streamline how authors should evaluate the strength of the evidence, the quality of the study design, and the relevance of the results to answering the research question [53–55]. In addition, subjective assessment of evidence plays a central role when evaluating qualitative research, for instance, to inform the development of guidelines and the formulation of policy [56,57].

Systematic guidelines help define the criteria for subjective evaluations, such as the relevance and adequacy of data, coherence of results, or methodological limitations of the study design. Such a standardized approach would be especially useful for many-analysts projects. Many-analysts projects share similarities with systematic literature reviews, as both require integrating multiple sources to address a single research question. We argue that analysis teams will be able to assess the evidence derived from their analysis more comprehensively if they use criteria similar to those used to assess evidence from randomized controlled trials, systematic reviews, and qualitative research.

### 1.3. Current study

The aim of the current project is twofold. First, we aimed to advance many-analysts studies by developing a subjective evidence evaluation survey (SEES). This survey includes aspects of evidence covered in the previous literature on subjective scientific assessments. Second, we aimed to develop a methodological and analytic strategy to effectively synthesize responses to the SEES.

The methods proposed here are particularly relevant for project leaders of many-analysts studies. Project leaders can use our methods to capture the beliefs of the analysis teams about the evidence for the hypothesized effect of interest more comprehensively. Furthermore, the SEES identifies potential methodological concerns of the analysis teams and may therefore safeguard against unwarranted certainty in drawing conclusions in many-analysts studies. Importantly, the SEES is intended to supplement—not supplant—objective measures of evidence such as the summary of outcome metrics.

In the following, we will describe the reactive-Delphi expert consensus procedure used in the collaborative development of the SEES. We then present the SEES and illustrate how to use it with responses from analysts in the Many-Analysts Religion Project. Appendix A provides more comprehensive guidance and detailed instructions for using the SEES. A Qualtrics template for the SEES is available on the OSF at: https://osf.io/axg2y.

## 2. Development of the subjective evidence evaluation survey

The idea for the SEES arose from the experience some of us (A.S. and S.H.) had in leading a many-analysts project (e.g. [23]), in which we felt we lacked the tools to fully and systematically represent the analysis teams’ efforts and insights that were privately communicated to us. To that effect, we considered which aspects of subjective evidence would be important to capture systematically and also agreed that the development of such a tool would only be successful if it was developed in collaboration with other experts. For these reasons, we decided to develop the SEES together with an expert panel in relevant scientific areas following a preregistered ‘reactive-Delphi’ expert consensus procedure [58] as implemented in [7] and [59]. The Delphi procedure iteratively determined the consensus of experts on the selection, wording, and content of items in multiple rounds. The development of the SEES included the creation of the initial item list, the consensus building using the Delphi method, and a final discussion round to finalize the survey.

### 2.1. Creating the initial item list

During the planning phase, authors A.S., S.H. and E.J.W. drafted an initial item list containing 22 items, which was based on checklist and guidance articles on systematic literature reviews [55] and evaluating qualitative evidence [57,60], and items used in previous many-analysts studies [21,23].^2^

### 2.2. Recruitment of the expert panel

On 25 November 2022, we contacted 93 experts, including project leaders of many-analysts and multiverse studies listed in [7], along with co-authors of the same publication. In addition, we reached out to experts in systematic literature reviews and evaluating qualitative evidence (e.g. co-authors of [53–57]). Furthermore, we invited measurement and general methodology experts, selecting them based on our knowledge of publications on cautionary notes and common pitfalls in scale construction, and on Bayesian methodology. Finally, we included experts recommended by fellow panel members. From the 93 experts, 45 agreed to participate in developing the SEES, seven declined our invitation, 38 did not respond to our request and three invitations bounced. Of these 45 experts, 37 finished all three consensus rounds.

### 2.3. Expert consensus procedure

We conducted a total of three rounds of rating by the Delphi method. In each round, the experts rated each item on a 9-point Likert-type scale ranging from 1 (Definitely not include this item) to 9 (Definitely include this item). Based on the panel responses, we iteratively refined our survey in each round by deleting, adding, or rewording items until we achieved consensus and support.

We preregistered a criterion that items with a median recommendation rating of 6 or higher and an interquartile range of 2 or smaller (indicating consensus) would be eligible for inclusion in the SEES (cf. [7,59]).^3^ This criterion was applied to all items except one. In round 3 of the expert consensus procedure, item 8 from the subjective evidence subscale received a median support rating of 8 but lacked consensus, with an interquartile range of 4. Despite the large interquartile range, we chose to add this item to the survey based on its high level of support. Specifically, the discussion round and feedback from the panel members suggested that the majority felt quite strongly that the item should be included as it addressed an aspect of evidence that was otherwise lacking (i.e. it was not covered by other items yet); 70% of the experts strongly endorsed the item and gave a score of 7–9 on the inclusion scale. All items received approval from panel members during the discussion round.

We also polled the panel on whether to offer a dichotomous response option or a Likert-type scale for each item in the SEES. The panel preferred a Likert-type scale. In the final version of the SEES, the items are answered using a 4-point Likert scale. The ideal number of response options is a topic of debate (see also [61] for a comprehensive review of the literature concerning survey and scale construction). When comparing 5-point Likert scales to 7-point Likert scales and 10-point Likert scales, some research suggests that a higher number of response options may possess better psychometric qualities (e.g. [62]), while others indicate that a lower number of answer options (5-point Likert scales versus 7-point Likert scales) yields comparable data quality (e.g. [63]). Ultimately, we chose to include a smaller number of answer options to keep the response options clear and interpretable. Additionally, we chose to omit a midpoint as a response option. Scales without midpoint and with a smaller number of answer options (i.e. 2 or 4) demonstrate in some research similar or slightly higher reliability compared to scales with a midpoint (e.g. [64]). Apart from considering psychometric properties, we also wanted to discourage participants from choosing the midpoint when they are undecided [65–67] or when an item is not applicable to their analyses. For such cases, we provided an ‘not applicable/I do not know’ answer option. A detailed description of the different stages of the Delphi method can be found at https://osf.io/jk674/.

## 3. The subjective evidence evaluation survey

The final version of the SEES consists of 18 items divided across two subscales asking (a) how their beliefs in the hypothesized effect of the study changed after their analyses (‘subjective evidence subscale’) and (b) whether they thought the methodology of the study was appropriate (‘methodological appropriateness subscale’).

The full survey is intended to be administered after analysis teams have conducted their analyses (but before they have seen other teams’ findings) and submitted their conclusions to the project leaders. In case analysis teams consist of multiple researchers, the survey should be filled out once per team.

### 3.1. Prior beliefs on plausibility

We recommend asking analysis teams how they evaluate the plausibility of the hypothesis of interest (i.e. item 1 of the subjective evidence subscale) *before having seen the data*. This not only provides valuable information on how the hypothesis is perceived, but also allows the project leaders to investigate confirmation bias (i.e. are prior beliefs related to reported outcomes?) and belief updating (i.e. are posterior beliefs related to reported outcomes and/or is the shift in beliefs related to the reported outcomes?). This item could be embedded in a questionnaire that captures the background and demographic information of the analysis teams (e.g. their expertise, academic position, familiarity with the topic).

1. Before having seen the data, do you find the hypothesized effect or relation plausible?

This item is answered on a 4-point Likert scale with response options ‘yes, definitely’, ‘yes, mostly’, ‘no, mostly not’, and ‘no, definitely not’. Project leaders can choose whether or not to include a ‘not applicable/I do not know’ option. This option is probably not necessary when assessing prior beliefs, but it could be added for consistency with the wording of items in the subjective evidence subscale.

### 3.2. Subjective evidence subscale

The subjective evidence subscale consists of eight questions, each accompanied by a short example (in italics) to illustrate the intended meaning. All items are answered on a 4-point Likert scale with response options ‘yes, definitely’, ‘yes, mostly’, ‘no, mostly not’, ‘no, definitely not’, and a ‘not applicable/I do not know’ option. Counter-indicative items (i.e. items 4, 5, 6 and 7 that indicate lower belief in the hypothesis) are to be reverse-coded. Analysis teams can provide additional feedback for each item in an open text box.

#### 3.2.1. Questions

SE_1 [Plausibility] Taking into account the results of your analyses, do you find the hypothesized effect or relation plausible? *For instance, obtaining substantial evidence that forcing a smiling facial position increases funniness ratings of cartoons shifts your beliefs on the facial feedback hypothesis from sceptical to favourable.*
SE_2 [Robustness] If applicable, is the hypothesized effect or relation consistent across all conducted analyses? *For instance, results from robustness checks or sensitivity analyses are consistent with the hypothesized effect found in the primary analysis.*
SE_3 [Evidence for effect] Does your analysis based on the observed data provide substantial evidence for the hypothesized effect or relation? *For instance, in a study on the recognition speed of words versus non-words, the confidence/credible interval of the effect size does not include zero.*
SE_4 [No evidence against effect*] Does your analysis based on the observed data provide substantial evidence *against* the hypothesized effect or relation? *For instance, evidence points in the opposite direction than hypothesized, or the evidence favours the null hypothesis.*
SE_5 [Subgroup homogeneity*] If applicable, does the hypothesized effect or relation vary between subgroups or data exclusion criteria? *For instance, a treatment benefited patients with moderate or severe depression but not patients with mild depression.*
SE_6 [Subconstruct homogeneity*] If applicable, does the hypothesized effect or relation vary for the different facets of the construct? *For instance, in a study on religiosity and well-being, religiosity was related to psychological and social well-being but not to physical well-being, that is, the relation is not stable across all measured facets of the variable well-being.*
SE_7 [No alternative explanations*] Do your analyses suggest plausible alternative explanations for the hypothesized effect or relation? *For instance, including socioeconomic status as a covariate eliminates the hypothesized relation between place of residence (rural versus urban) and happiness.*
SE_8 [Substantial effect size] Do you believe the size of the effect is substantial enough to be translated into real-life implications? *For instance, an effect of 2 points on a 7-point happiness scale might be perceived as having real-life consequences, whereas an effect of 0.1 points might not.*

### 3.3. Methodological appropriateness subscale

The methodological appropriateness subscale consists of 10 items, each accompanied by a short example (in italics) to illustrate the intended meaning. All items are answered on a 4-point Likert scale with response options ‘major concerns’, ‘moderate concerns’, ‘minor concerns’, ‘no concerns’, and a ‘not applicable/I do not know’ option. Analysis teams can provide additional feedback for each item in an open text box.

### 3.4. Questions

MA_1 [Sampling plan] Do you have concerns about the appropriateness of the sampling plan for the objectives of the research? *For instance, a study on global religiosity was conducted only in countries that are predominantly Christian which is a threat to external validity.*
MA_2 [Statistical power] Do you have concerns that the number of observations may not be sufficient to assess the hypothesized effect or relation? *For instance, there were not enough trials within participants or participants in conditions to reach sufficient statistical power.*
MA_3 [Missing values] Do you have concerns about missing values on the relevant variables? *For instance, there are too many missing values to draw a statistically valid conclusion, or the pattern of missing values appears non-random.*
MA_4 [Biased sample] Do particular sample characteristics (e.g. age, gender, socioeconomic status) raise concerns for the hypothesized effect or relation? *For instance, in a study on cognitive decline, the average age of the sample of older adults was relatively low (e.g. 60 years), which is a threat to generalizability across populations.*
MA_5 [Study setting] Do particular characteristics related to the setting of the study raise concerns for the hypothesized effect or relation? *For instance, a study on live social interactions was researched online, which is a threat to generalizability across contexts.*
MA_6 [Reliability] Do you have concerns about the reliability of the primary measures (i.e. measures producing similar results under consistent conditions)? *For instance, the measures were internally inconsistent, that is, results across items measuring a given construct were not consistent as indicated by Cronbach’s alpha.*
MA_7 [Validity] Do you have concerns about the validity of the measures (i.e. whether the measures capture the constructs of interest)? *For instance, a person’s level of social skills was measured by the number of friends they have, which is a threat to construct validity.*
MA_8 [Research design] Do you have concerns about the appropriateness of the research design for addressing the aims of the research? *For instance, a correlational study on obesity and depression was conducted to determine whether obesity causes depression.*
MA_9 [Missing variables] Do you have concerns that some necessary variables were missing to assess the hypothesized effect or relation? *For instance, a pre-intervention baseline measure, a control group, or important covariates were missing.*
MA_10 [Analysis] Do you have concerns about the appropriateness of your analysis for answering the research question? *For instance, some statistical assumptions were violated and could not be sufficiently addressed in the analysis.*

### 3.5. Computational model

The computational model we developed to synthesize responses to the SEES is based on cultural consensus theory [68–71]. Cultural consensus theory models are used in the analysis of response data where there is no ‘ground truth’ but the goal is to determine a collective opinion on a specific topic. Applied to the SEES for many-analysts studies, the cultural consensus theory model estimates the analysis teams’ collective opinion for each of the scale items, henceforth referred to as *consensus*, as well as overall, on the evidence in the data related to the research question.

We believe the Bayesian cultural consensus theory model is preferable to standard descriptive statistics such as sum scores or means given the structure of the data and desiderata for the results. First, the model is suited for ordinal data, which is not straightforward in many measurement/simple hierarchical models. Second, the model is relatively simple and closely related to well-known IRT models [69] such as the Graded Response Model [72] and the Item Factor Model [73]. Third, the model is hierarchical on the item level, assuming a shared ‘latent’ variable per subscale and taking into account similarities between analysis teams. Fourth, in addition to the hierarchical estimation of the consensus answer, the model also estimates a between-analysts parameter (i.e. scepticism, defined as an analyst’s tendency to select lower (versus higher) values on the response scale) and a between-item parameter (i.e. ‘difficulty’; whether the items elicit polarizing responses from the analysts) which may carry relevant information for understanding the results. Fifth, the Bayesian approach (a) enables estimation even with a small number of observations (which is likely in many-analysts projects), while the central limit theorem may fail to provide reliable estimates and (b) provides a quantification of uncertainty by means of the credible intervals [74–76]. Sixth, the model allows for extension to investigate different cultures of raters, which may be of interest in some many-analysts projects (e.g. compare evidence evaluation by theoretical experts to methodological experts).

Specifically, the applied computational model is an adapted version of the latent truth rater model proposed in [69] and extended in [76]. The model is implemented in the Stan programming language using the No-U-Turn sampler [77,78]. Appendix B contains a formal description of the model. In addition, appendix B contains a validation of the two-component structure of the SEES using Bayesian confirmatory factor analysis for ordinal data using the blavaan package in R [79].

### 3.6. An example application of the subjective evidence evaluation survey

To showcase the intended use of the SEES in a many-analysts project, we asked the analysis teams (*N* = 120) of the Many-Analysts Religion Project to retrospectively fill out the preliminary version of the SEES (i.e. the round 3 version of the expert panel procedure; see https://osf.io/4ypzv) based on their analysis for the project’s first research question: ‘Do religious people self-report higher well-being?’, approximately one year after the project had been completed. For this research question, all but three teams reported positive effect size estimates (standardized beta coefficients) with confidence/credible intervals excluding zero, suggesting a positive relation between religiosity and self-reported well-being in the dataset.

The SEES survey was completed by 42 analysis teams (35% of all analysis teams) and therefore these data cannot be taken to reflect the overall consensus from the Many-Analysts Religion Project. The sample of responders does not appear to be biased with regard to self-reported expertise and reported effect sizes. That is, the overall median and its median absolute deviation of the reported effect sizes in the sample of non-responders in the Many-Analysts Religion Project (0.114 [0.035]) are comparable to those of our subsample (0.129 [0.044]). Additionally, responders and non-responders are similar regarding the means and standard deviations of their self-reported methodological knowledge (*M* = 4.07, s.d. = 0.64 for responders versus *M* = 4.01, s.d. = 0.71 for non-responders) and substantive knowledge (*M* = 2.76, s.d. = 1.41 for responders versus *M* = 2.63, s.d. = 1.22 for non-responders). Nevertheless, these data merely serve as an illustration for how to use and analyse the SEES.

For each team, we assessed (1) the collective opinions for each survey item as well as the overall collective opinion for both subscales, (2) the change from prior to final beliefs about the plausibility of the effect, and (3) the correlations between the reported effect sizes and the prior beliefs, final beliefs, and the estimates of individual scepticism. Note that the Many-Analysts Religion Project analysis teams filled out a preliminary version of the SEES (i.e. the version after the third and final round of the consensus procedure) in which the subjective evidence subscale was phrased as statements rather than questions. In the final discussion round these items were reworded as questions rather than statements for consistency with the methodological appropriateness subscale. The response scale labels were changed from ‘strongly agree’, ‘somewhat agree’, ‘somewhat disagree’, and ‘strongly disagree’ to ‘yes, definitely’, ‘yes, mostly’, ‘no, mostly not’, ‘no, definitely not’. We avoided modifying other aspects of the items, including their content. As an illustration, the first item in the subjective evidence subscale (i.e. SE_1 [Plausibility]) was worded as follows in the preliminary version of the SEES provided to the 42 analysts: ‘Taking into account the results of my analyses: I find the hypothesized effect or relation plausible. *For instance, having obtained substantial evidence that forcing a smiling facial position increases funniness ratings of cartoons shifts your beliefs on the facial feedback hypothesis from skeptical to favourable*’.

#### 3.6.1. Subjective evidence

**Figure 1.**
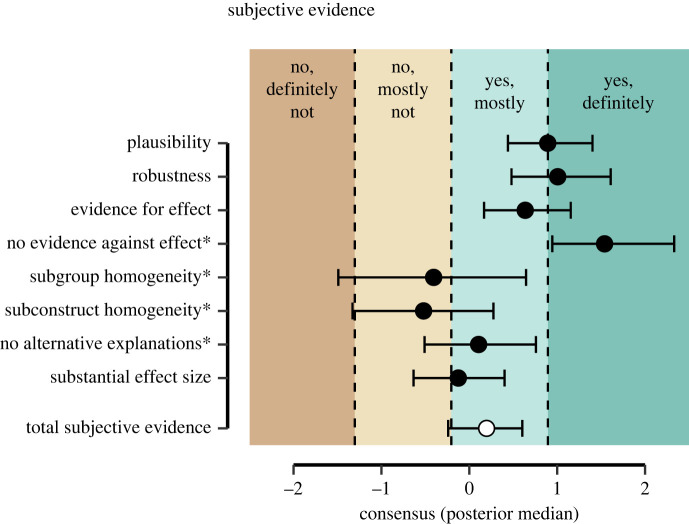
Estimated consensus for the subjective evidence subscale. The black points show the posterior medians (plus 95% credible interval) of the consensus, including the category thresholds. Items followed by an asterisk reflect items that have been reverse-coded and their labels have been changed for interpretability. The white marker at the bottom reflects the overall median assessment (plus 95% CI) of the subjective evidence subscale.

Figure 1 shows the model-based consensus for each item of the subjective evidence subscale, including the average response category thresholds and their labels (see also figure 11 for a depiction of the observed item ratings). The consensus represents the true location of the items on an assumed underlying unidimensional scale ranging from minus to plus infinity. When interpreting the consensus, it is crucial to note that they can only be interpreted in relation to the response category thresholds, which are represented by different colours in the figure. The posterior median and 95% credible interval of the overall consensus for the *subjective evidence* subscale is 0.20 [−0.24, 0.60] (visualized by the white marker in the figure) and thus largely falls into the response category ‘Yes, mostly’, and the standard deviation across items is 0.71. This indicates that the general consensus is that the analysis teams mostly believe that their analysis provides evidence for the hypothesis that religious people self-report higher well-being, with some variation across items. For instance, for item 4 (‘no evidence against effect’) the majority of analysis teams indicated that there is no evidence against the effect of interest (i.e. they indicated that ‘[their] analysis based on the observed data did definitely not provide substantial evidence *against* the hypothesized effect or relation’). This result is in line with the fact that all effect sizes were positive, which led the authors to conclude that in this dataset indeed there is a relation between religiosity and self-reported well-being.

For items 5 (‘subgroup homogeneity’) and 6 (‘subconstruct homogeneity’), the analysis teams seem neutral regarding whether effects differed across subgroup analyses and whether effects differed across different subconstructs. The large credible intervals for these items reflect the uncertainty in the estimated consensus, as more than half of the analysis teams considered the items not applicable (suggesting that they did not conduct subgroup analyses or subconstruct analysis). Despite the great uncertainty, however, these items contain noteworthy information. The teams that conducted these analyses seem relatively sceptical about the effect, especially compared to the other aspects of evidence. The cautious scepticism regarding subconstruct homogeneity aligns with the findings of the team leaders from the Many-Analysts Religion Project, as well as some of the published commentaries. Specifically, the project leaders indicated that the relationship between religiosity and well-being showed the strongest relationship when focusing on psychological well-being, followed by social well-being, and least with physical well-being [50]. This insight was gained through exploring the teams’ reported operationalizations of the dependent variable. However, scepticism regarding subgroup homogeneity had not been addressed by the project leaders. The implementation of SEES facilitates the identification of such nuanced insights that otherwise remain hidden and prompts team leaders to delve deeper into analytic results underlying sceptical responses.

#### 3.6.2. Methodological appropriateness

**Figure 2.**
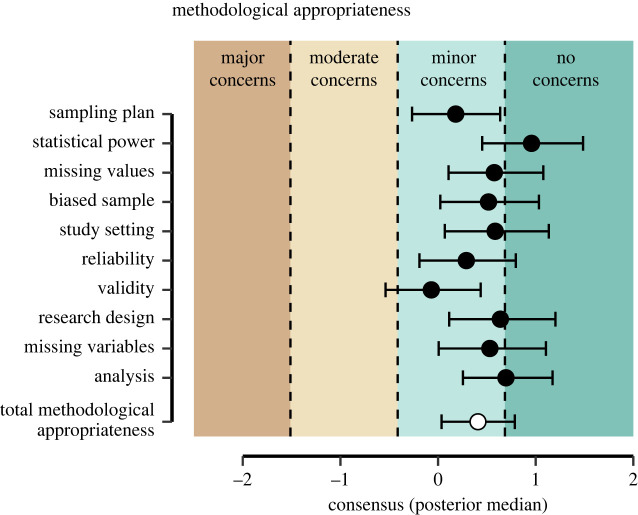
Estimated consensus for the methodological appropriateness subscale. The black points show the posterior medians (plus 95% credible interval) of the consensus, including the category thresholds. The white marker at the bottom reflects the overall median assessment (plus 95% CI) of the methodological appropriateness subscale.

Figure 2 shows the model-based consensus for each item of the 10 items of the methodological appropriateness subscale (see also figure 12 for a depiction of the observed item ratings). Compared to the subjective evidence subscale, the latent consensus for the methodological appropriateness subscale appears more similar. The posterior median of the overall consensus for the *methodological appropriateness* subscale is 0.41 with a 95% credible interval ranging from 0.04 to 0.79 and the standard deviation across items is 0.33. This indicates that the general consensus of the analysis teams is that there are minor to no methodological concerns regarding the analysis of the hypothesis that religious people self-report higher well-being. The posterior medians for almost all items reflect the analysis teams’ assessment of ‘minor concerns’; with the exception of item 2 (regarding the sufficiency of the number of observations) for which the posterior median reflects ‘no concerns’ (and perhaps item 10 on the analysis). For item 7 (validity), the analysis teams were most sceptical, with the credible interval of the consensus reaching an assessment of ‘moderate concerns’. This may reflect certain concerns raised in the published commentaries of the Many-Analysts Religion Project regarding measurement invariance [47,48], specifically, the observation that the religiosity construct does not maintain the same factor structure across all included countries.

#### 3.6.3. Subjective beliefs and effect size estimates

**Figure 3.**
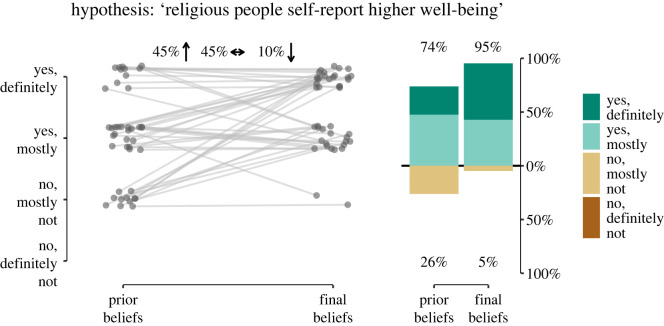
Prior and final beliefs about the plausibility of the hypothesis. The left side of the figure shows the change in beliefs for each analysis team. Forty-five per cent of the teams considered the hypothesis more likely after having analysed the data than prior to seeing the data, 10% considered the hypothesis less likely having analysed the data, and 45% did not change their beliefs. Plausibility was measured on a 4-point Likert scale ranging from ‘strongly disagree’ to ‘strongly agree’. Points are jittered to enhance visibility. The right side of the figure shows the distribution of the Likert response options before and after having conducted the analyses. The number at the top of the data bar indicates the percentage of teams that agreed that the hypothesis was plausible (in green) and the number at the bottom of the data bar (in brown/orange) indicates the percentage of teams that disagreed that the hypothesis was plausible.

Figure 3 displays the average prior and final beliefs about the plausibility of the hypothesis of interest.^4^ Researchers’ prior beliefs about religiosity being positively related to self-reported well-being were already high (*M* = 3.00 on the 4-point Likert scale), but were raised further after having conducted the analysis (*M* = 3.48 on the 4-point Likert scale). Specifically, before seeing the data, 73.81% of the teams considered it likely that religiosity is related to higher self-reported well-being. This percentage increased to 95.24% after having seen the data.

Following [21,23], we explored whether expectations and confirmation bias influenced the outcomes of the analysis teams and whether analysis teams updated their beliefs after conducting their analysis. To this aim, we assessed whether the reported effect sizes were positively related to the subjective assessments of the plausibility of the research question before and after analysing the data. In addition, we evaluated whether the effect sizes were related to the estimates of individual scepticism (i.e. their general tendency to select lower answer options on the scale; corresponding to the *β* parameters in the formal model description) on the subjective evidence subscale. Here, we would expect a *negative* correlation between effect size estimates and individual scepticism, reflecting that analysis teams who found lower effect sizes were subjectively more sceptical (less optimistic) about evidence for the research question. These hypotheses were tested against the null hypothesis that there is no relation between reported effect sizes and subjective beliefs or scepticism. As subjective beliefs were measured on a 4-point Likert scale, we used a rank-based Spearman correlation for the first two correlations and a Pearson correlation for the relation between effect size and estimated scepticism.

**Figure 4.**
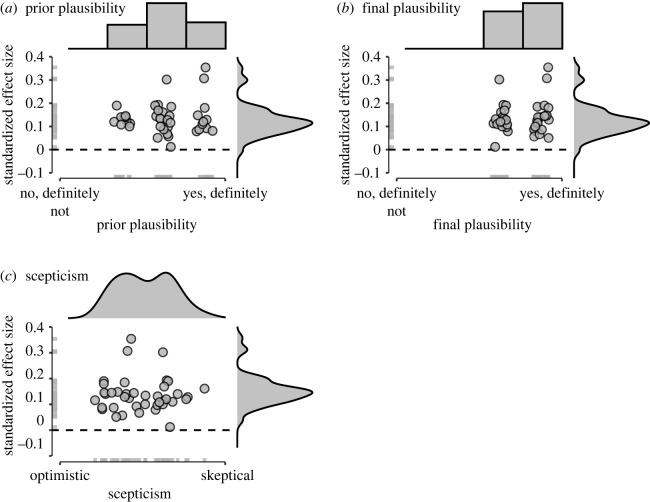
Reported effect sizes (beta coefficients) and subjective beliefs about the likelihood of the hypothesis. Panel (*a*) shows the relation between effect size and prior beliefs for the research question. Panel (*b*) shows the relation between effect size and final beliefs for the research question and panel (*c*) shows the relation between effect size and the analysis teams’ level of scepticism regarding the evidence. In (*a*,*b*), points are jittered on the *x*-axis to enhance visibility. The dashed line represents an effect size of 0. Histograms/density plots at the top represent the distribution of subjective beliefs and the density plots on the right represent the distribution of reported effect sizes.

**Figure 11.**
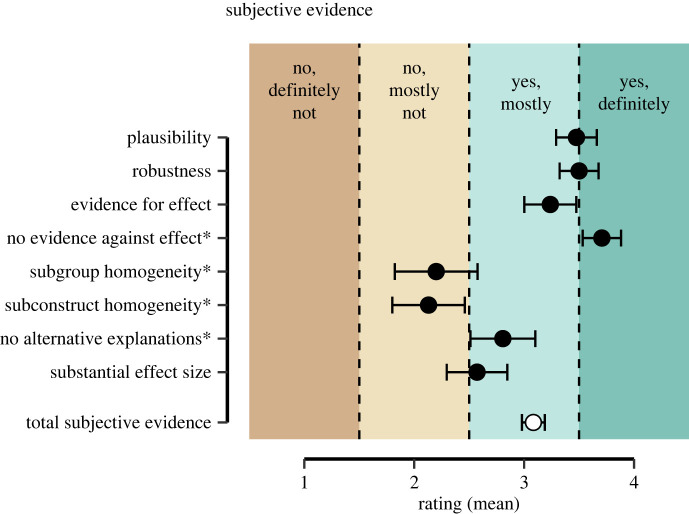
Observed item ratings for the subjective evidence subscale. The black points show the means (plus 95% confidence interval) of the item ratings, including the category thresholds. Items followed by an asterisk reflect items that were reverse-coded. The white point at the bottom reflects the overall mean assessment (plus 95% confidence interval) of the subjective evidence subscale.

**Figure 12.**
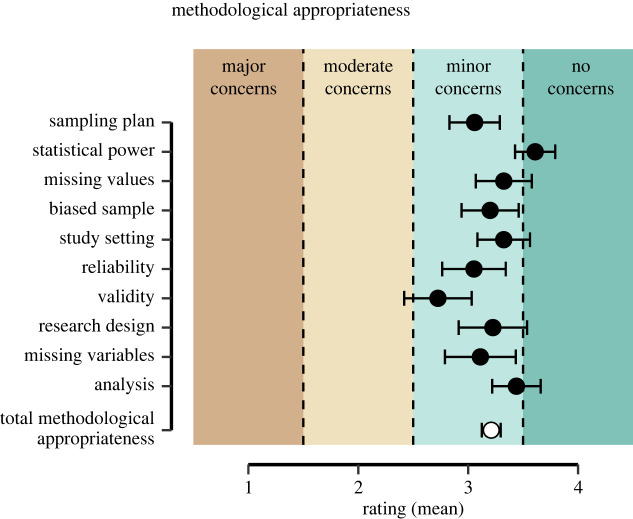
Observed item ratings for the methodological appropriateness subscale. The black points show the means (plus 95% confidence interval) of the item ratings, including the category thresholds. The white point at the bottom reflects the overall mean assessment (plus 95% confidence interval) of the methodological appropriateness subscale.

The correlations are visualized in figure 4. We obtained moderate evidence *against* a positive relation between prior beliefs about the plausibility of the hypothesis and the reported effect sizes: BF_+0_ = 0.13; BF_0+_ = 7.94, *ρ*_*s*_ = −0.11, 95% credible interval [−0.38, 0.21]. In addition, we found strong evidence against a positive relation between posterior beliefs about the plausibility of the research question and the reported effect sizes: BF_+0_ = 0.08; BF_0+_ = 12.11, $\rho_{s}=-0.27,95\%$ credible interval [−0.53, 0.06].^5^ Finally, we found anecdotal evidence against a negative relation between estimated scepticism on the SEES and reported effect sizes: BF_+0_ = 0.35; BF_0+_ = 2.85, ρ=0.00,95% credible interval [−0.30, 0.28].

As mentioned in [23], these results provide no indication that expectations and confirmation bias influenced the teams’ results (i.e. prior beliefs are not related to reported effect sizes), nor do they provide evidence for belief updating after having conducted the analyses (i.e. posterior beliefs are not related to the reported effect sizes). Note, however, that the updating of beliefs may not have happened because prior beliefs about research question 1 were already in line with the outcomes; most teams expected and reported evidence for a positive relation between religiosity and well-being, with little variation between teams in both beliefs and reported effect sizes. This lack of variability across teams may also underlie the absence of a correlation between individual differences in objectively reported effect sizes and estimated scepticism. In cases where the analysis teams report diverging results (i.e. conclusions that are qualitatively different) one may expect to find stronger belief updating and larger variability in individual scepticism.^6^

Moreover, the long time period between conducting the analyses and completing the SEES prevents strong interpretations of these results. Instead, as mentioned at the beginning of this section, the data presented here should be regarded merely as a demonstration of the intended use of the SEES.

## 4. Discussion

The present work introduces the SEES as a tool to systematically explore and quantify subjective measures of evidence in many-analysts projects. The development of the SEES was informed by work on systematic reviews and qualitative research and was collaboratively developed by 37 experts in related fields in a reactive-Delphi procedure, reflecting a consensus among these experts. The 18-item survey covers various aspects of evidence, such as coherence, robustness, and relevance as well as diverse methodological concerns regarding the underlying design and data that may affect the interpretation of the obtained statistical results.

The first aim of the current project was to develop a measurement tool to capture analysts’ beliefs about the evidence obtained in a many-analysts project. Combined with the objective outcomes of the many-analysts approach such as effect size estimates or proportion of statistically significant results, the SEES contributes to a comprehensive summary of the obtained evidence for the hypothesis of interest in a many-analysts project. By capturing analysts’ beliefs about the evidence of the hypothesis of interest, the SEES presents a solution to a challenging task: bringing insights and concerns of the analysis teams to the surface in a systematic and scalable manner.

Rather than requiring each team to write a narrative evaluation, project leaders can have them complete the SEES to extract a collective assessment of insights and concerns from all participating teams. Here, we suggested to have the SEES completed once per analysis team. However, if the (additional) goal is to identify potential within-team variability, project leaders may consider eliciting one answer per analyst. This approach may require an extension to the proposed cultural consensus theory model to account for dependencies of analysts within teams.

The SEES introduces several advantages over previously employed methods for subjectively assessing evidence in many-analysts projects. First, in past projects, only a limited number of items concerning subjective evidence (e.g. plausibility of the effect) were administered to analysis teams, primarily to evaluate confirmation bias and belief updating [21]. By contrast, the SEES encompasses a comprehensive list of aspects of subjective evidence and methodological appropriateness deemed important by experts. Second, prior many-analysts studies did not gather information on how analysts themselves rated the appropriateness of their analysis; instead, if analyses were evaluated, the quality of analysis was typically rated by other participating analysts. The SEES allows analysts to reflect on the appropriateness of their own analysis, which can either replace or supplement time-consuming peer assessments. Third, by including the methodological appropriateness subscale, the SEES also offers teams a platform to indicate the quality of the provided dataset and research design. The insights gained from the SEES can encourage team leaders to perform additional analyses (e.g. subgroup or subconstruct analyses) that add important nuances to the main results, as seen in our example application. At minimum, it compels the project leads to reflect on the methodological concerns indicated by the analysts.

Importantly, we do not advocate replacing objective measures of evidence with subjective measures. The subjective measures of evidence complement the objective measures by putting the findings in perspective and/or highlighting inconsistencies in the results or flaws in the research design. The subjective evaluation captured by the SEES provides concrete input for the general discussion of a many-analysts manuscript. In addition, answers to the SEES might reveal potential sources of variability in the obtained results; for instance, teams that investigated different subgroups might reach different conclusions and obtain a different outcome metrics from teams that only targeted one large group.

The second aim of the current project was to outline an analytic strategy for interpreting SEES outcomes, quantifying belief updating in analysis teams, and connecting outcomes of the SEES with objective outcome measures. Concretely, this strategy allows project leaders to investigate whether prior expectations or confirmation bias influenced the results (cf. [21]). We recommend using the outlined Bayesian cultural consensus theory model to analyse the SEES data, but also acknowledge that our analysis strategy is not necessary when employing the SEES. Instead, project leaders may opt to calculate sum scores per subscale and/or overall sum scores for the entire survey (see the appendix for a visualization of the results from the example application based on descriptive statistics).

We contribute to the current literature about guidelines on many-analysts studies [7] by offering concrete advice on how to analyse and interpret (part of) the data obtained in many-analysts projects. This, together with advancements on synthesizing objective outcome metrics across analyses based on the same data (e.g. [36,37]), can move the field beyond drawing conclusions based on (visual) inspection of the analysts’ outcomes.

### 4.1. Limitations

The applicability of the SEES may vary depending on the nature of the many-analysts project. In a typical scenario, many-analysts projects leave some room for analytic flexibility and subjective principled decisions. The nuances that arise from this subjectivity can then be captured by the SEES. There might be situations, however, where many-analysts projects essentially eliminate opportunity for analysts to choose which variables to assess. For instance, commentaries published on the Many-Analysts Religion Project (e.g. [42,49]) argued that project leaders should have reduced analytic variability arising from differences in the interpretation of the research question. For instance, instead of asking ‘Do religious people report higher well-being?’ it would be more beneficial to ask whether ‘*specific* behaviours and/or beliefs benefit *specific* populations’ well-being or health in *specific* contexts’ [42, p. 2]. In a many-analysts project which aims to reduce analytic variability stemming from different interpretations of the research question as much as possible, capturing the subjective evaluations of the analysis teams may not be advised.

Analysis teams in the Many-Analysts Religion Project, however, viewed the exploration of subgroup effects or of testing of the effect across different sub-constructs as integral to answering the research question. The results from a more generally formulated research question may therefore be more typical of the heterogeneity in the literature on a particular effect. After all, an important motivation to conduct a many-analysts study is to capture (the consequences of) different principled decisions throughout the analytic process.

In addition, some datasets or research designs may render some items irrelevant. For instance, the item on reliability may be irrelevant if the data only feature single item measures. In [21], for instance, the dependent variable was the number of red cards given to dark skinned soccer players. This variable is a single-item measure in which the reliability (e.g. internal consistency) is irrelevant and the SEES item may confuse the participating teams. Although the analysis teams can always indicate ‘not applicable/I do not know’, the project leaders may also choose to remove these items from the survey.

When multiple research questions are posed, participating teams may find it cumbersome to answer the SEES for each question. If the research questions are answered based on one dataset, rather than on multiple datasets (e.g. stemming from multiple experiments), project leaders could present the methodological appropriateness subscale just once to the teams.

Finally, we invited experts from previous many-analysts studies and experts in the field of systematic literature reviews and qualitative research. However, the framing of the items and the examples given may speak more to researchers from the social and behavioural sciences than to researchers from other areas. If necessary, project leaders from many-analysts studies could use the SEES flexibly and reword the examples to better fit the specific field of study.

It should be noted that if project leaders use a modified version of the SEES (e.g. removing the reliability item if it is irrelevant in their study), it should not be presented as reflecting a consensus approach because removing items may influence the estimation accuracy of the proposed cultural consensus theory model. The performance of the computational model also depends on the number of participating analysis teams with more precise estimation of consensus as the number of teams increases. In simulation studies, we found good model performance based on visual inspection for a sample of *N* = 42—that is, the sample size in our example application—and recommend using at least that many responses when applying the proposed computational model to the SEES. We also found satisfactory model performance based on visual inspection for 20 analysis teams, although with less favourable recovery for true consensus (i.e. wider posterior distributions) compared to the larger sample. The full results can be accessed in the electronic supplementary material at https://osf.io/4cesj.

### 4.2. Concluding thoughts

In the current project, we collected pilot data to illustrate the intended use and analysis of the SEES, by asking analysts from the Many-Analysts Religion Project to retroactively complete the survey. An obvious next step would be to implement the SEES in a future many-analysts project. We hope to have inspired project leaders of many-analysts projects to consider adding subjective evidence assessment to their future projects and thereby allowing for a more complete evaluation of the outcomes.

## 5. Disclosures

### 5.1. Preregistration

Prior to collecting data, we preregistered the full procedure for the reactive Delphi method to develop the SEES on the Open Science Framework at https://doi.org/10.17605/OSF.IO/E4QNY. Any deviations from the preregistration are mentioned in this paper. Note that we also preregistered a procedure for collecting SEES data from the 2023 cohort of a graduate course. However, since only two teams of students decided to participate, we could not continue this line of data collection. Instead, we decided to contact the analysts from the Many-Analysts Religion Project again and ask them to retroactively fill out the SEES. This latter approach was not preregistered.

### 5.2. Ethical approval

The expert consensus procedure was approved by the local ethics board of the University of Amsterdam (registration no. 2022-PML-15535). All participants were treated in accordance with the Declaration of Helsinki. Experts who participated in all consensus rounds and approved the final version of the manuscript were given the opportunity to become co-authors of this publication. The collection of pilot data to illustrate how many-analysts studies can be analysed with the SEES was approved by the local ethics board of the University of Amsterdam (registration number: FMG-4376). Researchers who participated in the pilot study received an €8 voucher as compensation.

## Figures and Tables

**Figure 5.**
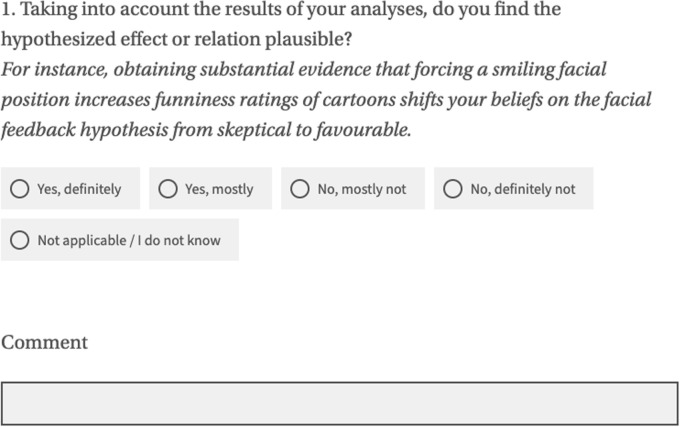
Example of the presentation of the subjective evidence subscale, item 1.

**Figure 6.**
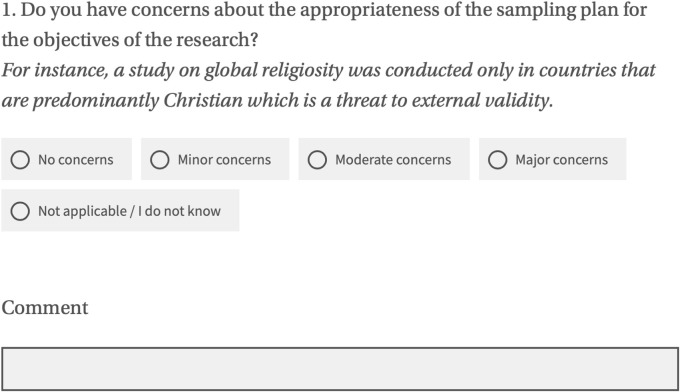
Example of the presentation of the subjective evidence subscale, item 1.

**Figure 7.**
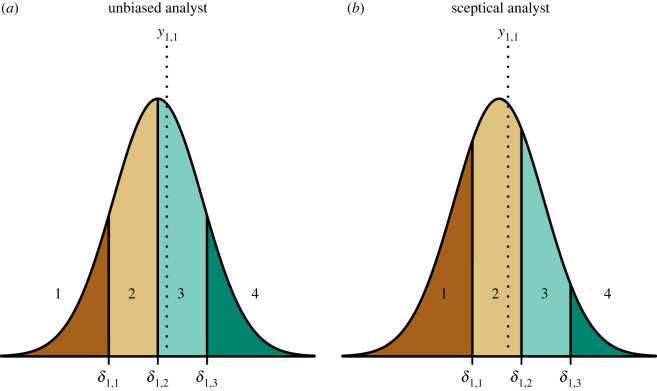
Illustrating the relationship between latent probability distribution over the predicted Likert scores, item appraisal, and decision criteria. In a hypothetical scenario, two analysts with the same item appraisal *y*_*r*,*i*_ differ in their individual decision criteria *δ*_*r*,*c*_, influenced by a shift parameter. For this particular example, the unbiased analyst (*β* = 0; shown in *a*) would respond ‘yes, mostly’, while the sceptical analyst (*β* > 0.5; shown in *b*) would respond ‘no, mostly not’.

**Figure 8.**
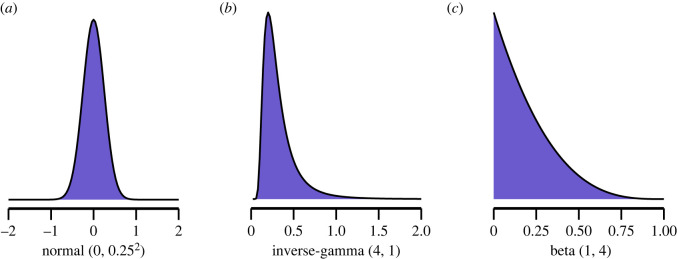
Visualization of the group-level mean prior distributions (*a*), the group-level standard deviation prior distributions (*b*) and the prior distribution on the applicability probabilities (*c*) used in the SEES model.

**Figure 9.**
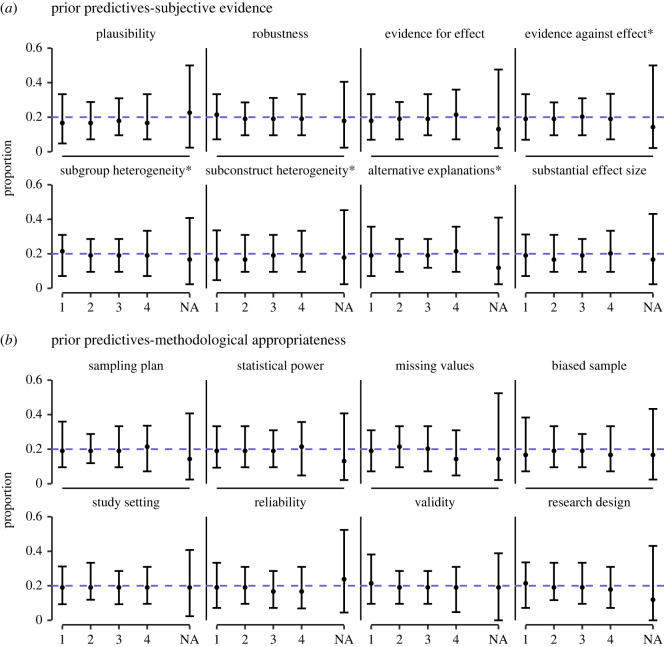
Prior model predictions of data for both subscales. Before having been in contact with the data, the model predicts analysts will select each response category nearly equally often, showing a slightly lower tendency for the ‘not applicable/I do not know’ response category.

**Figure 10.**
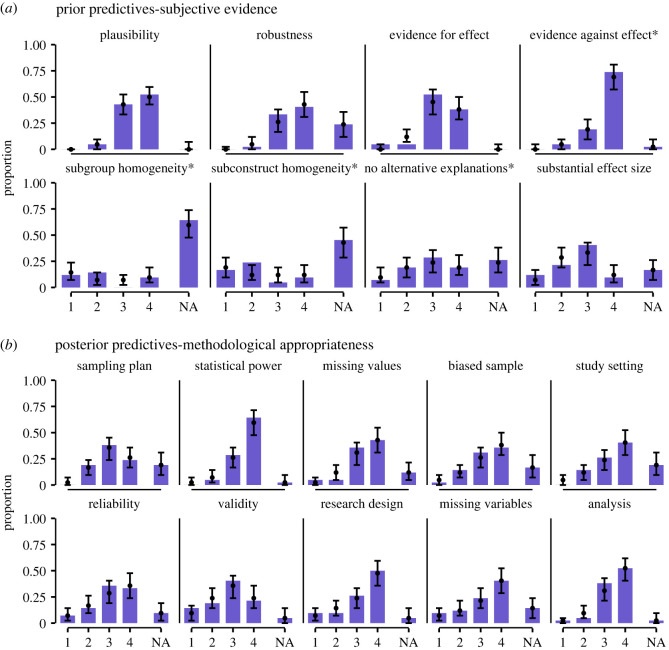
Posterior model predictions of data for both subscales. For each item, the purple bars reflect the observed relative proportion of responses and the black dots plus 95% credible interval reflect the predicted responses from the model.

**Figure 13.**
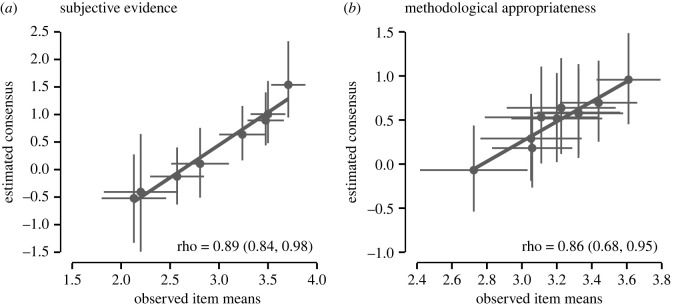
Correlation between the estimated consensus and the observed item means for the subjective evidence subscale (*a*) and the methodological appropriateness subscale (*b*). Vertical error bars reflect the 95% credible interval of the estimated consensus and horizontal error bars reflect the 95% confidence interval of the observed scores. ‘rho’ gives the Bayesian Spearman correlation between the estimated consensus and observed means.

## Data Availability

Readers can access the preregistration, the materials for the study, the data and the R code to conduct all analyses (including all figures) in our OSF folder at: https://osf.io/jk674/ [80]. The data are provided in electronic supplementary material [81].

## Footnotes

^1^
Alternative approaches for synthesizing outcomes in many-analysts projects (e.g. considering only the sign of the effect size; focusing on evidential measures such as *p*-values or Bayes factors) do not seem satisfactory, especially when quantifying the size of the effect is essential (e.g. [51]).

^2^
The initial item list can be accessed via https://osf.io/jk674/.

^3^
Note that a median rating of 6 is lower than the typical median rating of 8 in medical sciences, where Delphi procedures are frequently applied. We adopted a more lenient cutoff as methodological recommendations for the social and behavioural sciences differ somewhat from those aimed at reaching a medical consensus, where even slight hesitation warrants caution.

^4^
Note that prior beliefs about the plausibility of the effect were reported on a 7-point scale instead of a 4-point scale in the Many-Analysts Religion Project. To make these prior beliefs compatible with the posterior plausibility assessment as included in the SEES (item 6), we recoded the 7-point scale into a 4-point scale: 1 became 1, 3 became 2, 5 became 3 and 7 became 4. Responses in the in-between categories were randomly assigned; 2 was randomly assigned to become 1 or 2, 4 was randomly assigned to become 2 or 3, and 6 was randomly assigned to become 3 or 4.

^5^
In the sample of non-responders from the Many-Analysts Religion Project, we reach the same conclusions regarding the absence of evidence for confirmation bias. That is, we found strong evidence *against* a positive relation between prior beliefs about the plausibility of the hypothesis and the reported effect sizes BF_+0_ = 0.08; BF_0+_ = 12.23, *ρ*_*s*_ = −0.12, 95% credible interval [−0.34, 0.12]. Regarding the updating of beliefs we found inconclusive evidence in the sample of non-responders. The relation between posterior beliefs about the plausibility of the research question and the reported effect sizes was BF_+0_ = 1.09; BF_0+_ = 0.91, *ρ*_*s*_ = 0.19, 95% credible interval [−0.03, 0.38].

^6^
For the second research question discussed in the Many-Analysts Religion Project, analysis teams reported more qualitatively different results compared to the first research question. And indeed, in this case, we found strong evidence for belief updating, that is, posterior beliefs were positively correlated with reported effect sizes [23].

## Appendix A. Subjective evidence evaluation survey: instructions to project leaders

The SEES has been developed to facilitate and extend the interpretation of evidence for a given research hypothesis in many-analysts projects. In a many-analysts project, multiple research teams are invited to independently analyse the same data and address the key research question [6,21]. The core idea of a many-analysts approach is to demonstrate the range of justifiable analytic decisions and their consequences in terms of outcomes and conclusions, thereby unveiling the robustness or fragility of the effect of interest. Results from different analysis teams are typically summarized by means of effect size estimates (e.g. odds ratios or beta weights). The SEES has been developed to complement and extend this method, by allowing analysis teams to subjectively reflect on (a) the evidence that their analysis provides for the hypothesis of interest and (b) the quality of the materials and the data. This allows the analysis teams to communicate a more fine-grained evaluation of the evidence obtained through their analysis, yet in a structured manner.

The SEES consists of two subscales, eliciting analysis teams to answer questions about (a) how analysts’ beliefs in the hypothesized effect of the study changed after their analyses (subjective evidence subscale) and (b) whether they thought the methodology of the study was appropriate (methodological appropriateness subscale). The full survey should be administered after analysis teams conducted and submitted their analyses. In addition, in order to assess belief updating, item 1 of the subjective evidence subscale should also be administered *prior* to receiving the to-be-analysed data. In case analysis teams consist of multiple researchers, the survey should be filled out once per team.

**A.1. Pre-analysis phase**

We recommend asking analysis teams to evaluate the plausibility of the hypothesis of interest *before having seen the data*. This not only provides valuable information on how the hypothesis is perceived, but also allows the project leaders to investigate confirmation bias (i.e. are prior beliefs related to reported outcomes?) and belief updating (i.e. are posterior beliefs related to reported outcomes and/or is the shift in beliefs related to the reported outcomes?). This item could be embedded in a questionnaire on the background of analysis teams (e.g. expertise, academic position, familiarity with the topic).

1. Before having seen the data, do you find the hypothesized effect or relation plausible?

Answer options: ‘yes, definitely’, ‘yes, mostly’, ‘no, mostly not’, and ‘no, definitely not’. Project leaders can choose whether or not to include a ‘not applicable/I do not know’ option. This option is probably not necessary for the pre-analysis survey, but it could be added for consistency with the post-analysis survey.

**A.2. Post-analysis phase**

**A.2.1. Subjective evidence subscale**

The subjective evidence subscale consists of eight items. Each item contains the question of interest plus a short example to illustrate the intended meaning (in italics). All items are answered on a 4-point Likert scale with response options ‘yes, definitely’, ‘yes, mostly’, ‘no, mostly not’, ‘no, definitely not’ and a ‘not applicable/I do not know’ option. Counter-indicative items (i.e. items indicating lower belief in the hypothesis) are to be reverse-coded (i.e. item 4, 5, 6 and 7). Analysts can provide additional feedback for each item in an open text box. An example of how the items and response options could be presented is shown in figure 5.

**A.3. Instructions**

‘Please answer the following questions about your assessment of the *evidence* based on your analysis for [research question]. We understand that this is subjective; there are no correct or wrong answers. Hypothesis: [the hypothesis of interest]. Please base your answers on your interpretation of the analysis conducted by your team’.

**A.4. Questions**

1. Taking into account the results of your analyses, do you find the hypothesized effect or relation plausible? *For instance, obtaining substantial evidence that forcing a smiling facial position increases funniness ratings of cartoons shifts your beliefs on the facial feedback hypothesis from sceptical to favourable.*
2. If applicable, is the hypothesized effect or relation consistent across all conducted analyses? *For instance, results from robustness checks or sensitivity analyses are consistent with the hypothesized effect found in the primary analysis.*
3. Does your analysis based on the observed data provide substantial evidence for the hypothesized effect or relation? *For instance, in a study on the recognition speed of words versus non-words, the confidence/credible interval of the effect size does not include zero.*
4. Does your analysis based on the observed data provide substantial evidence *against* the hypothesized effect or relation? *For instance, evidence points in the opposite direction than hypothesized, or the evidence favours the null hypothesis.*
5. If applicable, does the hypothesized effect or relation vary between subgroups or data exclusion criteria? *For instance, a treatment benefited patients with moderate or severe depression but not patients with mild depression.*
6. If applicable, does the hypothesized effect or relation vary for the different facets of the construct? *For instance, in a study on religiosity and well-being, religiosity was related to psychological and social well-being but not to physical well-being, that is, the relation is not stable across all measured facets of the variable well-being.*
7. Do your analyses suggest plausible alternative explanations for the hypothesized effect or relation? *For instance, including socioeconomic status as a covariate eliminates the hypothesized relation between place of residence (rural versus urban) and happiness.*
8. Do you believe the size of the effect is substantial enough to be translated into real-life implications? *For instance, an effect of 2 points on a 7-point happiness scale might be perceived as having real-life consequences, whereas an effect of 0.1 points might not.*

**A.4.1. Methodological appropriateness subscale**

The methodological appropriateness subscale consists of 10 items. Each item contains the question of interest plus a short example to illustrate the intended meaning (in italics). All items are answered on a 4-point Likert scale with response options ‘major concerns’, ‘moderate concerns’, ‘minor concerns’, ‘no concerns’, and a ‘not applicable/I do not know’ option. Analysis teams can provide additional feedback for each item in an open text box. An example of how the items and response options could be presented is shown in figure 6.

**Instructions.** ‘Please answer the following questions about your assessment of *methodological concerns* regarding your analysis for [research question]. We understand that this is subjective; there are no correct or wrong answers. Hypothesis: [hypothesis of interest]. Please base your answers on your reflections regarding the provided data and study design’.

**Questions.**

1. Do you have concerns about the appropriateness of the sampling plan for the objectives of the research? *For instance, a study on global religiosity was conducted only in countries that are predominantly Christian which is a threat to external validity.*
2. Do you have concerns that the number of observations may not be sufficient to assess the hypothesized effect or relation? *For instance, there were not enough trials within participants or participants in conditions to reach sufficient statistical power.*
3. Do you have concerns about missing values on the relevant variables? *For instance, there are too many missing values to draw a statistically valid conclusion, or the pattern of missing values appears non-random.*
4. Do particular sample characteristics (e.g. age, gender, socio-economic status) raise concerns for the hypothesized effect or relation? *For instance, in a study on cognitive decline, the average age of the sample of older adults was relatively low (e.g. 60 years), which is a threat to generalizability across populations.*
5. Do particular characteristics related to the setting of the study raise concerns for the hypothesized effect or relation? *For instance, a study on live social interactions was researched online, which is a threat to generalizability across contexts.*
6. Do you have concerns about the reliability of the primary measures (i.e. measures producing similar results under consistent conditions)? *For instance, the measures were internally inconsistent, that is, results across items measuring a given construct were not consistent as indicated by Cronbach’s alpha.*
7. Do you have concerns about the validity of the measures (i.e. whether the measures capture the constructs of interest)? *For instance, a person’s level of social skills was measured by the number of friends they have, which is a threat to construct validity.*
8. Do you have concerns about the appropriateness of the research design for addressing the aims of the research? *For instance, a correlational study on obesity and depression was conducted to determine whether obesity causes depression.*
9. Do you have concerns that some necessary variables were missing to assess the hypothesized effect or relation? *For instance, a pre-intervention baseline measure, a control group, or important covariates were missing.*
10. Do you have concerns about the appropriateness of your analysis for answering the research question? *For instance, some statistical assumptions were violated and could not be sufficiently addressed in the analysis.*

**A.5. Background information**

**A.5.1. Survey development**

The SEES was developed in collaboration with 37 experts in relevant scientific areas following a preregistered ‘reactive-Delphi’ expert consensus procedure [58] as implemented in [7,59]. Selected areas of expertise included many-analysts and multiverse studies, systematic literature reviews, questionnaire development, and general methodology. Over three rounds, experts were asked to rate each item in the SEES on a 9-point Likert-type recommendation scale ranging from 1 (*Definitely do not include this item*) to 9 (*Definitely include this item*). Based on the panel responses, the survey was iteratively refined in each round by deleting, adding, or rewording items until achieving consensus and support.

We considered items to have reached panel consensus if the interquartile range of expert ratings was 2 or smaller, and we regarded items as having obtained panel support if their median ratings were 6 or higher. Note that we preregistered that only items with a median recommendation rating of 6 or higher and an interquartile range of 2 or smaller would be eligible for inclusion in the SEES. This criterion was applied to all items except one. In round 3 of the expert consensus procedure, item 8 from the subjective evidence subscale received a median support rating of 8 but lacked consensus, with an interquartile range of 4. Despite this, we chose to add this item to the survey. In the discussion round, some panel members explicitly approved the items; none of the panel members objected to them.

**A.5.2. Adaptations**

We encourage project leaders from many-analysts studies to use the SEES flexibly and reword the examples for the items to the specific field of study if necessary. In addition, some datasets or research designs may render some items irrelevant and may confuse the participating teams. Although the analysis teams can always indicate ‘not applicable/I do not know’, the project leaders may also choose to remove these items from the survey. Finally, when analysis teams have answered multiple research questions based on one dataset, rather than on multiple datasets (e.g. stemming from multiple experiments), project leaders could present the methodological appropriateness subscale only once to the teams.

**A.5.3. Proposed analysis**

The SEES data can be analysed in a cultural consensus theory model [68–71,76], which allows one to synthesize responses and capture the collective opinion about the subjective evidence in many-analysts projects. A comprehensive description of the cultural consensus theory model applied to the SEES can be found in appendix B.

## Appendix B. Cultural consensus theory model

Here, we describe the adapted version of the latent truth rater model [69,76] which we use to synthesize responses and capture collective opinions on the two subscales of the SEES.

**B.1. Model description**

Within the latent truth rater model the variable *x*_*r*,*i*,*s*_ denotes the observed and discrete responses provided by analyst *r* for item *i* on subscale *s*.^7^ For convenience, we will drop the subscript *s* in further descriptions. The variable *x*_*r*,*i*_ takes on the value *x*_*r*,*i*_ = 0 when the response to an item is ‘not applicable/I do not know’. In all other cases, for each item, it takes on one of the *C* = 4 Likert scores. For instance, in the subjective evidence subscale, *x*_*r*,*i*_ = 1 corresponds to the analyst’s response of ‘no, definitively not’, *x*_*r*,*i*_ = 2 to ‘no, mostly not’, *x*_*r*,*i*_ = 3 to ‘yes, mostly’, and *x*_*r*,*i*_ = 4 corresponds to ‘yes, definitely’.

The model determines three factors that influence the observed responses. The first factor is the applicability of the item which is captured by the probability *π*_*i*_. Higher values of *π*_*i*_ indicate higher non-applicability resulting in more analysts selecting the ‘not applicable/I do not know’ option. The remaining responses are influenced by the second and third factors. The second factor, the latent appraisal for the item *y*_*r*,*i*_, is a combination of item properties, such as the latent consensus and the item difficulty (i.e. extent of eliciting polarizing responses). The third factor relates to individual characteristics of the analysts, that is, their individual bias, which determines their decision criteria, denoted as *δ*_*r*,*c*_.

The latent truth rater model assumes that each item has some latent consensus (originally termed ‘item truth’ *θ*_*i*_ among analysts—their true collective opinion—on an abstract psychological scale, e.g. the conceived plausibility of the hypothesized effect or relation). Given that an analyst has sufficient knowledge to answer the item, the following process is assumed to take place. Across all items, the analyst draws a mental sample for their decision criteria *δ*_*r*,*c*_. Then, for each item, they draw a mental sample for their item appraisal *y*_*i*,*r*_. The analysts’ responses are then determined by where the latent appraisal *y*_*i*,*r*_ falls in relation to their decision criteria *δ*_*c*,*r*_. The analyst responds with the next higher response category if a latent appraisal for a particular item exceeds a decision criterion, as illustrated in figure 7 and explained mathematically below:

$$x_{r,i}=\left\{ \begin{array}{cc} 1\; & \text{ if }y_{r,i}\leq \delta_{r,1}\;\\ c=2\text{ or }3\; & \text{ if }\delta_{r,c-1}<y_{r,i}<\delta_{r,c}\;\\ 4\; & \text{ if }\delta_{r,3}<y_{r,i}.\; \end{array}\right.$$

**B.2. Latent consensus**

The appraisal of an item *y*_*r*,*i*_ comprises two latent components: the latent consensus (*θ*_*i*_) and the item difficulty (*κ*_*i*_). Within the context of a many-analysts project, the primary focus is on estimating the latent consensus as it represents the items’ true location on an assumed underlying unidimensional scale. The assumption is that, depending on the item difficulty, the latent consensus is reflected by different analysts with varying degrees of accuracy. In other words, items that elicit polarizing responses from the analysts (i.e. items with lower inter-rater reliability) will be estimated with lower accuracy compared to items that the majority of analysts agree on.

In a scenario where we can estimate the latent consensus without any confounding factors, the item difficulty would be *κ*_*i*_ = 1, implying that all analysts have the identical information necessary to respond to the item.

**B.3. Response bias**

As with the item appraisal, the latent decision criteria *δ*_*r*,*c*_ for each analyst likewise consist of two components: a shift parameter *β*_*r*_ and the response thresholds *λ*_*c*_:

$$\delta_{r,c}=\lambda_{c}+\beta_{r}.$$

The shift parameter determines an analyst’s tendency to select lower or higher values on the response scale (i.e. representing individual scepticism). The response thresholds, unaffected by biases, are determined solely by the number of response categories and are identical across all items and analysts, that is, *λ*_*c*_ = logit(*c*/*C*). In the scenario of an entirely unbiased analyst, their shift parameter would be *β*_*r*_ = 0, suggesting a neutral inclination toward selecting values on the response scale (see figure 7*a*). For shift parameters greater than zero, the individual decision criteria for each item move to the right, leading to lower values on the response scale and consequently more sceptical responses. To illustrate this shift, see figure 7*b* which depicts the latent probability distribution across predicted Likert scores for a sceptical analyst with a shift parameter of *β* = 0.5. Conversely, when *β*_*r*_ < 0, the decision criteria shift to the left, leading to more positive responses.

Bringing together all components of the model, the probabilities of selecting a specific response category can be modelled using the cumulative distribution of the standard logistic distribution, given by *F*(*x*) = (1 + e^−*x*^)^−1^. Given the latent appraisal *y*_*r*,*i*_ and the latent decision criteria *δ*_*r*_, the responses *x*_*r*,*i*_ then follow an ordered logistic distribution:

$$P(x_{r,i}|y_{r,i},\delta_{r})=\left\{ \begin{array}{cc} 1-F(y_{r,i}-\delta_{r,1})\; & \text{ if }x_{r,i}=1\;\\ F(y_{r,i}-\delta_{r,c-1})-F(y_{r,i}-\delta_{r,c})\; & \text{ if }1<x_{r,i}<4\;\\ F(y_{r,i}-\delta_{r,3})\; & \text{ if }x_{r,i}=4.\; \end{array}\right.$$

**B.4. Prior distributions**

We based our prior distributions on the suggestions provided in [76] as a starting point. Subsequently, we refined the values to achieve prior predictions that reflect reasonable response patterns (i.e. an approximately uniform distribution of the predicted responses) but are still vague enough to ensure proper updating of the parameters in light of the data. A visualization of the prior distributions for the group-level means and group-level standard deviations and the prior distribution on the applicability probability are visualized in figure 8.

The applicability probability *π*_*i*_ for each item is assumed to be drawn from a beta distribution that mildly favours items being considered appropriate. The remaining parameters are assumed to be drawn from normal distributions. Due to identifiability constraints discussed in [76], the group-level mean for the item difficulty *κ* is fixed to 1:

$$\begin{array}{rl} \pi_{i} & ∼\text{beta}\;(1,4)\\ \theta_{i} & ∼\text{normal}\;(\mu_{\theta },\sigma_{\theta }^{2})\\ \kappa_{i} & ∼\text{normal}\;(1,\sigma_{\kappa }^{2})\\ \mathrm{and}\;\beta_{r} & ∼\text{normal}\;(\mu_{\beta },\sigma_{\beta }^{2}). \end{array}$$

The group-level means for the consensus and the shift parameter are chosen to be relatively uninformative. Their values are drawn from a normal distribution centred at 0 with standard deviations that favour values centred around zero:

$$\mu_{\theta }∼\text{normal}\;(0,0.25^{2})$$

and

$$\mu_{\beta }∼\text{normal}\;(0,0.25^{2}).$$

The standard deviations are drawn from inverse-gamma distributions which allow for moderate heterogeneity for the consensus and individual biases:

$$\begin{array}{rl} \sigma_{\theta } & ∼\text{inverse-gamma}\;(4,1)\\ \sigma_{\kappa } & ∼\text{inverse-gamma}\;(4,1)\\ \mathrm{and}\;\;\sigma_{\beta } & ∼\text{inverse-gamma}\;(4,1). \end{array}$$

**B.5. Assumptions**

The model is based on several key assumptions. First, it assumes that the probabilities of items being deemed non-applicable are independent across items. For instance, in the subjective evidence subscale, an analyst may feel insufficiently informed to respond to item 5 (*If applicable, does the hypothesized effect or relation vary between subgroups or data exclusion criteria?’*) and consequently select the response option ‘not applicable/I do not know’. However, this response may not affect whether they answer ‘not applicable/I do not know’ to item 3 (*Does your analysis based on the observed data provide substantial evidence for the hypothesized effect or relation?*). A violation of this assumption may overestimate the heterogeneity of the estimated applicability probabilities due to the absence of hierarchical shrinkage. Note that this independence assumption is only made for ‘not applicable/I do not know’ answer options. For the remaining answer options items and participants are assumed to be related through the hierarchical structure of the model parameters.

Second, the original version of the latent truth rater model included two additional parameters: a parameter describing an analyst’s inclination towards extreme responses (i.e. answering more confidently) and a parameter describing the conformity of an analyst to the group opinion. The conformity parameter describes the extent to which an analyst deviates from the group opinion—perhaps due to adopting an unconventional analytic approach. In other words, non-conforming analysts are more prone to give responses that differ from what we would anticipate based solely on the consensus. The current model assumes that analysts have no bias towards extreme responses and that there is a perfect alignment of opinions among analysts. The reason for these assumptions lies in the nature of the SEES, which by design, produces scarce data (with only 8 and 10 data points per analyst for each subscale, respectively) limiting its capacity to capture parameters characterizing each individual analyst. By placing additional assumptions on the extremity bias and group conformity, we were able to reduce the number of parameters and improve the model’s ability to recover true parameter values in a scarce data environment.

Third, following the recommendations in [76], the model assumes that an analyst’s decision criteria can be effectively described using only the shift parameter, eliminating the need to estimate each threshold separately. Lastly, the model places a sum-to-one constraint on the response thresholds *λ*_*c*_ so that for an unbiased analyst the model predicts *a priori* a uniform distribution over the survey responses. Assumptions three and four resolve identification issues within the model.

**B.6. Model validation**

To ensure the model aligns with theoretical expectations, we generated prior predictive plots for a sample of *n* = 42 which matches our pilot study’s sample size. In the absence of information regarding the evidence supporting a hypothesis, the methodological appropriateness of the research design, or the analysts responding to the SEES, we would expect a uniform distribution of responses. That is, we would expect *a priori* that each response category gets selected equally often. The prior predictive distributions confirm this expectation. Figure 9 presents the prior predictions for both subscales across a hypothetical group of 42 analysts. The response categories 1 to 4 are distributed approximately equally for each item, while the ‘not applicable/I do not know’ response category is anticipated to be chosen slightly less frequently.

The posterior predictive distribution can be interpreted as the model’s attempt to re-describe the behavioural data and constitutes another step in the model validation process. Predictions from an adequate model should resemble the behavioural data. In figure 10, we visualize for each item the relative proportion of observed responses from our pilot study (purple bars) and the model’s predicted responses (black dots and 95% credible intervals). Indeed, the posterior model predictions are able to re-describe the observed data.

In addition to examining prior and posterior predictive distributions, we ensured that the proposed computational model as well as the implemented Markov chain Monte Carlo (MCMC) algorithm is able to recover the prior distribution when no data are observed, that the implemented MCMC algorithm returns unbiased estimates, and that the data effectively update the prior beliefs. These additional checks were proposed in [81], based on the recommendations in [82,83]. We conducted these checks for samples of size *n* = 42 and *n* = 20 with satisfactory model performance. The full results can be accessed in the electronic supplementary material.

## Appendix C. Additional results

**C.1. Descriptive results**

Here, we report the findings of the example data from the Many-Analysts Religion Project using descriptive results, as an alternative to the modelling approach outlined in the main text.

**C.1.1. Subjective evidence**

The mean of the overall consensus rating for the *subjective evidence* subscale is 3.08; the 95% confidence interval is [2.98, 3.19]. Figure 13*a* shows the correlation between the observed item means and estimated consensus per item, indicating high correspondence between the observed and estimated item metrics (*ρ* = 0.89, 95% CI [0.84, 0.98]).

**C.1.2. Methodological appropriateness**

The mean of the overall consensus rating for the *methodological appropriateness* subscale is 3.21; the 95% confidence interval is [3.12, 3.3]. Figure 13*b* shows the correlation between the observed item means and estimated consensus per item, again indicating high correspondence between the observed and estimated item metrics (*ρ* = 0.86, 95% CI [0.68, 0.95]).

**C.2. Confirmatory factor analysis**

To validate the two-component structure of the SEES, we conducted Bayesian confirmatory factor analysis for ordinal data using the blavaan package in R [79]. Specifically, we fitted the measurement model assuming the eight *subjective evidence* items to load on one factor and the ten *methodological concerns* items to load on a second factor (covariance between factors: *ρ* = 0.47). All standardized factor loading was above 0.5 except for the ‘subgroup homogeneity’ (0.35) and ‘subconstruct homogeneity’ (0.17) items of the subjective evidence scale.

Following recommendations by [84,85], we applied model comparison using the ratio of differences in the leave-one-out cross-validation metric and its standard error to interpret evidence for the two-factor model over the null model (i.e. the independence baseline model). In particular, we found that the two-factor model fitted the data better than the baseline model, with a magnitude of 4.87 SE in LOO differences, which can be considered substantial [85].
